# Ultrasound-Assisted Extraction of Free and Bound Phenolics from Hawthorn (*Crataegus azarolus*) Leaves and their Antioxidant and Antidiabetic Activities

**DOI:** 10.1007/s11130-026-01505-0

**Published:** 2026-05-16

**Authors:** Esra Esin, Semra Topuz Türker, Mustafa Bayram, Cemal Kaya

**Affiliations:** Faculty of Engineering and Architecture, Department of Food Engineering, Tokat Gaziosmanpasa University, Tokat, 60250 Türkiye

**Keywords:** Free and bound phenolic compounds, Ultrasound-assisted extraction, Response surface methodology, Antioxidant activity, Antidiabetic potential

## Abstract

Hawthorn (*Crataegus azarolus*) leaves are an underutilized plant resource with potential as a source of bioactive phenolic compounds. This study investigated the ultrasound-assisted extraction (UAE) of free and bound phenolics from hawthorn leaves and evaluated their functional properties. The extraction parameters were optimized using response surface methodology (RSM) to maximize the total phenolic content (TPC) and total flavonoid content (TFC). Under optimal conditions, the free phenolic content reached 78.61 mg GAE g^− 1^ dry weight (DW), whereas the bound phenolic content reached 13.56 mg GAE g^− 1^ DW, accounting for approximately 15% of the total phenolic content. The free phenolic fraction exhibited higher antioxidant activity than the bound fraction in the ABTS, DPPH, and FRAP assays. Both fractions inhibited α-amylase and α-glucosidase, demonstrating their in vitro enzyme inhibitory potential. These results highlight the importance of evaluating both the free and matrix-bound phenolic fractions, and they support the potential use of hawthorn leaves as a natural source of functional ingredients.

**Supplementary Information:**

The online version contains supplementary material available at 10.1007/s11130-026-01505-0.

## Introduction

Natural products, particularly medicinal plants and herbs, have been widely used for the treatment of various diseases since prehistoric times. In recent years, interest in plant-based therapies has increased significantly due to their potential health benefits and lower side effects compared to synthetic drugs [1]. Among these, species of the *Crataegus* genus (hawthorn) have attracted considerable attention due to their rich bioactive composition [2].

The medicinal properties of hawthorn were first documented in Europe in the 19th century, particularly for their beneficial effects on cardiovascular diseases [3]. Beyond cardiovascular applications, hawthorn has traditionally been used for the treatment of kidney stones, gastrointestinal disorders, respiratory problems, cancer, neurological conditions, and hypercholesterolemia. Previous studies have demonstrated that hawthorn extracts exhibit antiinflammatory, antihypertensive, and antithrombotic activities [1, 4]. Despite the extensive use of hawthorn fruits, other plant parts such as leaves remain relatively underutilized, although they represent a potentially valuable source of bioactive compounds. The valorization of such underutilized plant materials may contribute to sustainable resource use and the development of value-added functional ingredients [5, 6].

The biological activity of hawthorn is largely attributed to its rich phenolic composition [7]. Phenolic compounds are widely distributed secondary metabolites that play a crucial role in plant defense mechanisms and exhibit strong antioxidant and therapeutic properties [8]. Hawthorn contains various phenolic acids, including chlorogenic, caffeic, ferulic, gallic, syringic, and *p*-coumaric acids, as well as flavonoids such as quercetin, rutin, apigenin, vitexin, and isovitexin [9, 10]. In addition, compounds such as anthocyanidins, proanthocyanidins, epicatechin, procyanidin oligomers, and cyanidin have also been identified [11].

Phenolic compounds in plant materials exist in both free and bound forms. Free phenolics are generally soluble and can be easily extracted, whereas bound phenolics are covalently linked to cell wall components such as cellulose, lignin, and proteins, making their extraction more challenging and often requiring hydrolysis [12]. Importantly, bound phenolics can constitute a substantial proportion of total phenolics in foods. For example, 50–95% of phenolics in fruits, 80–90% of phenolic acids in rice, and a significant portion in legumes are present in bound form [13–15]. There is no single standardized extraction method for free and bound phenolic compounds from plant materials due to their structural diversity and varying interactions with the plant matrix [16].

Efficient extraction of phenolic compounds is a critical step for their recovery and application. While conventional techniques such as maceration and decoction are widely used, ultrasound-assisted extraction (UAE) has gained attention due to its higher efficiency, reduced solvent consumption, and shorter extraction time [17]. However, extraction efficiency is influenced by several factors, including temperature, extraction time, solvent composition, and the chemical nature of the target compounds [18].

Hawthorn has been extensively investigated for its phenolic composition and biological activities. Existing studies have primarily focused on free phenolic fractions, while bound phenolics from hawthorn leaves remain largely unexplored. In this context, hawthorn leaves can be considered an underutilized plant material with potential for value-added applications. To the best of our knowledge, this study is the first to systematically extract, optimize, and comparatively evaluate both free and bound phenolic compounds from hawthorn leaves using alkaline hydrolysis [19, 20] combined with UAE technique. This integrated approach enables efficient recovery of bound phenolics and provides a more comprehensive assessment of the total phenolic potential of this plant material. Moreover, the valorization of such underutilized biomass may contribute to more sustainable use of plant resources and the development of novel functional ingredients. Furthermore, the simultaneous evaluation of antioxidant and in vitro antidiabetic activities under optimized conditions offers a functional perspective that has not been previously reported for hawthorn leaves. Therefore, this study aims to (i) optimize UAE conditions for free phenolics and alkaline extraction combined with UAE for bound phenolics, (ii) compare the extraction efficiency and phenolic profiles of both fractions, and (iii) evaluate their antioxidant and enzyme inhibitory activities.

## Materials and Methods

The materials and methods section is provided as an online resource (supplementary material).

## Results and Discussion

### Extraction and Optimization of Free Phenolic Compounds from Hawthorn Leaves

In this study, the effects of extraction time (X_1_: 5–90 min), temperature (X_2_: 30–70 °C), and ethanol (EtOH) concentration (X_3_: 10–90%) on TPC and TFC were investigated for the UAE of free phenolic compounds from hawthorn leaves (Table 1). The TPC values of the extracts ranged from 24.70 to 79.37 mg GAE g^− 1^ DW, while TFC values ranged from 20.91 to 61.82 mg QE g^− 1^ DW. Both TPC and TFC values were highest in trial 14, whereas the lowest values were observed in trial 10, indicating that the extraction conditions applied in trial 14 were more effective in recovering phenolic compounds. This variation suggests that the extraction parameters significantly influenced phenolic yield, potentially due to differences in solvent efficiency, extraction time, or temperature.

**Table 1:** Experimental design and obtained TPC and TFC values for free phenolic extraction using UAE

Trial no	Extraction time (min)	Temperature (°C)	EtOH concentration (%)	TPC (mg GAE g^− 1^ DW)	TFC (mg QE g^− 1^ DW)
1	47.50	50.00	50.00	71.45 ± 1.81^b*^	57.40 ± 0.84^b^
2	47.50	50.00	50.00	70.97 ± 2.64^b^	55.73 ± 1.73^bc^
3	47.50	30.00	90.00	51.68 ± 2.03^fg^	45.06 ± 1.13^g^
4	90.00	30.00	50.00	57.22 ± 2.96^de^	51.46 ± 1.00^e^
5	90.00	50.00	10.00	35.87 ± 2.29^j^	28.50 ± 1.85^j^
6	47.50	50.00	50.00	69.41 ± 2.96^b^	52.76 ± 1.47^de^
7	90.00	50.00	90.00	55.76 ± 1.87^de^	51.61 ± 2.27^e^
8	5.00	70.00	50.00	60.99 ± 2.90^c^	49.24 ± 1.65^f^
9	47.50	70.00	10.00	40.89 ± 2.23^h^	29.16 ± 1.70^j^
10	47.50	30.00	10.00	24.70 ± 1.51^l^	20.91 ± 0.53^k^
11	47.50	50.00	50.00	68.14 ± 2.47^b^	54.12 ± 0.54^cd^
12	5.00	30.00	50.00	58.58 ± 1.75^cd^	48.72 ± 1.74^f^
13	47.50	70.00	90.00	54.08 ± 1.27^ef^	52.24 ± 0.90^de^
14	90.00	70.00	50.00	79.37 ± 2.43^a^	61.82 ± 0.83^a^
15	47.50	50.00	50.00	68.08 ± 1.49^b^	53.42 ± 1.12^de^
16	5.00	50.00	90.00	49.43 ± 2.40^g^	41.13 ± 1.91^h^
17	5.00	50.00	10.00	28.64 ± 1.73^k^	21.26 ± 1.22^k^

*Small letters in the same column show difference between samples (*p* < 0.05)

Results are given as mean ± standard deviation (*n*=6)

*TPC* Total phenolic compound, *TFC* Total flavonoid compound, *GAE* Gallic acid equivalent, *QE* Quercetin equivalent

The TPC and TFC values of the hawthorn leaf extracts obtained under 17 different conditions showed significant variations (*p* < 0.05). Therefore, data optimization is required. The second-order polynomial models developed for the extraction processes and obtained by regression analysis during optimization are given by Eqs. 1 and 2 for TPC and TFC, respectively.

1 $$\begin{array}{c}TPC\left(mg\;GAE/g\;DW\right)=-15.75-0.04X_1+0.85X_2+1.99X_3\\+5.8\times10^{-3}X_1X_2-1.30\times10^{-4}X_1X_3-4.31\times10^{-3}X_2X_3\\-1.66\times10^{-3}X_1^2-6.45\times10^{-3}X_2^2-1.51\times10^{-2}X_3^2\end{array}$$

2 $$\begin{array}{c}TFC\left(mg\;QE/g\;DW\right)=3.57+0.01X_1+0.13X_3+1.37X_3\\+2.89\times10^{-3}X_1X_2+4.75\times10^{-4}X_1X_3-3.30\times10^{-4}X_2X_3\\-8.60\times10^{-4}X_1^2-8.20\times10^{-4}X_2^2-1.10\times10^{-2}X_3^2\end{array}$$

The effects of process variables on TPC and TFC during free phenolic compound extraction from hawthorn leaves are presented in the ANOVA table (Table S5). The quadratic models developed for both responses were statistically significant at the 99% confidence level (*p* < 0.01), and the lack of fit was statistically insignificant at the 95% confidence level (*p* > 0.05). A statistically insignificant lack of fit indicates that the developed model adequately describes the experimental data. In this study, the insignificant lack of fit (*p* > 0.05) confirms the suitability of the second-order polynomial models developed for TPC and TFC. In addition to the lack of fit values, the adequacy of the models in explaining the experimental data was evaluated using regression coefficient (R²), adjusted regression coefficient (Adj-R²), adequate precision, PRESS, and coefficient of variation (C.V. %) (Table S5).

The data analysis revealed that the linear effects of extraction time (X_1_), temperature (X_2_), and EtOH concentration (X_3_) were statistically significant (*p* < 0.05) at the 95% confidence level in the models developed for both TPC and TFC in the UAE process. The interaction effects exhibited distinct patterns of statistical significance: the time-temperature (X_1_X _2_) interaction was statistically significant for both TPC and TFC, whereas the time-EtOH concentration (X_1_X_3_) interaction was not statistically significant for either response. In contrast, the temperature-EtOH concentration (X_2_X_3_) interaction was statistically significant for TPC but not for TFC (Table S5).

The quadratic terms showed variable statistical significance where X_1_^2^ and X_2_^2^ were statistically significant for TPC but not for TFC whereas X_3_^2^ was statistically significant for both TPC and TFC. The UAE results for both TPC and TFC showed R^2^ and Adj-R^2^ values (Table S5) close to 1.00, indicating a strong correlation between the predicted and actual values. These results confirm the reliability of the proposed models for optimizing phenolic compound extraction from hawthorn leaves.

The 3D response surface plots (Fig. S1 and S2) show the effects of temperature, extraction time, and EtOH concentration on the TPC and TFC values of hawthorn leaf extracts obtained by UAE. At a fixed temperature (50 °C), increasing EtOH concentration enhanced TPC and TFC values up to an optimum level, after which a decline was observed. This suggests that EtOH–water mixtures are more effective than single solvents because they facilitate the extraction of compounds with different polarities. Polyphenols containing multiple hydroxyl groups generally exhibit hydrophilic characteristics and therefore show higher solubility in hydroalcoholic mixtures than in pure alcohol solvents [21]. A similar trend was observed for the interaction between EtOH concentration and temperature at a fixed extraction time (47.75 min). This behavior can be explained by Fick’s second law of diffusion, which states that solute concentrations in the solid matrix and solvent phase approach equilibrium after a certain period. Moreover, prolonged extraction may promote the reabsorption of phenolic compounds onto the plant matrix, thereby limiting further increases in phenolic yield. Consequently, extending extraction time beyond the optimal range does not significantly improve phenolic recovery [22].

In Figure S3, the experimental data for TPC and TFC are compared with the predicted values from the polynomial models, showing that the data points align closely along the 45° line. This indicates the suitability of both models.

In the optimization of TPC and TFC extraction from hawthorn leaves using UAE, the Design-Expert 7.0 software predicted the optimal extraction conditions. Based on the desirability function approach, four similar solution points were obtained (Table S6). Among these, 90 min extraction time, 70 °C temperature, and 55.33% EtOH concentration were selected as the optimal conditions. Under these conditions, the predicted TPC and TFC values were 78.61 mg GAE g^− 1^ DW and 63.95 mg QE g^− 1^ DW, respectively. Verification experiments performed in triplicate yielded values of 79.77 mg GAE g^− 1^ DW for TPC and 64.15 mg QE g^− 1^ DW for TFC. A one-sample t-test showed no statistically significant difference between the predicted and experimental values (*p* > 0.05).

### Extraction and Optimization of Bound Phenolic Compounds from Hawthorn Leaves

The effects of NaOH concentration (X_1_: 1–4 M), hydrolysis time (X_2_: 5–90 min), and temperature (X_3_: 30–70 °C) on TPC and TFC were investigated for the extraction of bound phenolic compounds from hawthorn leaves. The TPC values of the extracts ranged from 1.46 to 13.16 mg GAE g^− 1^ DW, while the TFC values varied between 0.81 and 9.77 mg QE g^− 1^ DW (Table 2). Both TPC and TFC values were highest in trial 15, whereas the lowest values were observed in trial 4, indicating that stronger alkaline conditions combined with appropriate hydrolysis time and temperature enhanced the release of bound phenolics. This can be attributed to the cleavage of ester and ether linkages between phenolic compounds and cell wall components under alkaline hydrolysis. In contrast, the lower yields observed in trial 4 suggest that insufficient hydrolysis conditions limited the release of bound phenolics, highlighting the critical role of process parameters in maximizing extraction efficiency.

**Table 2:** Experimental design and obtained TPC and TFC values for bound phenolic extraction using UAE

No	NaOH concentration (M)	Hydrolysis time (min)	Temperature (°C)	TPC (mg GAE g^− 1^ DW)	TFC (mg QE g^− 1^ DW)
1	1.00	47.50	30.00	6.42 ± 0.12^g^	4.62 ± 0.21^h^
2	1.00	90.00	50.00	8.06 ± 0.15^f^	6.25 ± 0.22^g^
3	2.50	47.50	50.00	12.42 ± 0.30^b^	8.43 ± 0.19^c^
4	2.50	5.00	30.00	1.46 ± 0.08k	0.81 ± 0.04^m^
5	2.50	5.00	70.00	1.94 ± 0.06^j^	1.37 ± 0.05^l^
6	2.50	47.50	50.00	11.39 ± 0.26^c^	8.07 ± 0.08^d^
7	2.50	90.00	30.00	9.22 ± 0.24^e^	7.41 ± 0.23^f^
8	4.00	5.00	50.00	5.28 ± 0.09^h^	4.30 ± 0.09^j^
9	4.00	47.50	30.00	9.33 ± 0.21^e^	6.45 ± 0.25^g^
10	4.00	90.00	50.00	12.74 ± 0.17^b^	9.30 ± 0.16^b^
11	1.00	5.00	50.00	1.64 ± 0.21^jk^	1.23 ± 0.05^l^
12	2.50	90.00	70.00	11.43 ± 0.30^c^	7.90 ± 0.07^de^
13	2.50	47.50	50.00	10.68 ± 0.35^d^	7.57 ± 0.23^f^
14	1.00	47.50	70.00	5.24 ± 0.07^h^	3.41 ± 0.19^k^
15	4.00	47.50	70.00	13.16 ± 0.33^a^	9.77 ± 0.18^a^
16	2.50	47.50	50.00	11.35 ± 0.09^c^	7.67 ± 0.36^ef^
17	2.50	47.50	50.00	11.24 ± 0.37^c^	8.05 ± 0.26^d^

*Small letters in the same column show difference between samples (*p* < 0.05)

Results are given as mean ± standard deviation (n=6)

*TPC* Total phenolic compound, *TFC* Total flavonoid compound, *GAE* Gallic acid equivalent, *QE* Quercetin equivalent

Second-order polynomial models developed for the extraction processes and obtained through regression analysis during the optimization study are given by Eqs. 3 and 4 for TPC and TFC, respectively.

3 $$\begin{array}{c}TPC\left(mg\;GAE/g\;DW\right)=-10.65+1.49X_1+0.24X_2\\+0.38X_3+4.08\times10^{-3}X_1X_2+4.17\times10^{-2}X_1X_3+5.06\times\\10^{-4}X_2X_3-0.44X_1^2-1.94\times10^{-3}X_2^2-4.74\times10^{-3}X_3^2\end{array}$$

4 $$\begin{array}{c}TFC\left(mg\;QE/g\;DW\right)=-7.43+0.42X_1+0.18X_2+0.28X_3\\-5.00\times10^{-5}X_1X_2+3.77\times10^{-2}X_1X_3-2.30\times10^{-5}X_2X_3\\-0.22X_1^2-1.21\times10^{-3}X_2^2-3.49\times10^{-3}X_3^2\end{array}$$

The effects of process variables on TPC and TFC values during the extraction of bound phenolic compounds from hawthorn leaves are presented in the ANOVA table (Table S7). The quadratic models developed for both responses were statistically significant at the 99% confidence level (*p* < 0.01), while the lack of fit was statistically insignificant at the 95% confidence level (*p* > 0.05). The R^2^ and Adj-R^2^ values of the models for TPC and TFC were very close to each other (Table S7), indicating that the models adequately explained the experimental data without including statistically insignificant terms. In addition, the low C.V. (%) confirms the high precision and reliability of the experimental data. Based on these findings, the proposed models were suitable for optimizing and predicting the extraction of bound phenolic compounds from hawthorn leaves.

The data analysis showed that the linear effects of NaOH concentration (X_1_) and hydrolysis time (X_2_) were statistically significant (*p* < 0.05) for both TPC and TFC, whereas the linear effect of temperature (X_3_) was statistically significant for TPC but not statistically significant for TFC in the UAE. The interaction effects exhibited distinct patterns of statistical significance: NaOH concentration-hydrolysis time (X_1_X_2_) and hydrolysis time-temperature (X_2_X_3_) interactions were not statistically significant for either TPC or TFC, whereas NaOH concentration–temperature (X_1_X_3_) interaction was statistically significant for both responses (Table S7).

The quadratic terms showed variable statistical significance: X_1_^2^ was statistically significant for TPC (*p* < 0.05) but not statistically significant for TFC (*p* > 0.05), whereas X_2_^2^ and X_3_^2^ were statistically significant for both TPC and TFC (*p* < 0.05). The UAE method showed R^2^ and Adj-R^2^ values (Table S7) close to 1.00 for both TPC and TFC, indicating a strong correlation between the predicted and actual values. These results confirm the reliability of the proposed models for optimizing phenolic compounds extraction from hawthorn leaves.

The 3D response surface plots in (Fig. S4 and S5) illustrate the effects of NaOH concentration (M), hydrolysis time (min) and temperature (°C) on the TPC and TFC values of the hawthorn leaves extracts using UAE. Analysis of the interaction between time and NaOH concentration at a fixed temperature (50 °C, the midpoint of the temperature range) revealed that increasing NaOH concentration led to an increase in TPC and TFC values. However, beyond a certain threshold, a decline was observed. Similarly, the effects of NaOH concentration and temperature on TPC and TFC values at a fixed extraction time (47.50 min) mirrored the trends observed under fixed temperature conditions.

Figure S6 compares the experimental values of TPC and TFC with the values predicted by the polynomial models. The data were distributed along line 45, indicating the reliability and suitability of both models.

In the optimization study aimed at maximizing TPC and TFC extraction from hawthorn leaves using UAE, the Design-Expert 7.0 software predicted the optimal extraction conditions. Based on the desirability function approach, 13 closely related solution points were obtained (Table S8). Among these, 3.87 M NaOH concentration, 83.35 min extraction time, and 48.24 °C temperature were selected as the optimal conditions. Under these conditions, the predicted TPC and TFC values were 13.56 mg GAE g^− 1^ DW and 9.92 mg QE g^− 1^ DW, respectively. Verification experiments performed in triplicate yielded values of 13.31 mg GAE g^− 1^ DW for TPC and 9.77 mg QE g^− 1^ DW for TFC. A one-sample t-test showed no statistically significant difference between the predicted and experimental values (*p* > 0.05).

In literature, Alirezalu et al. [23] reported that the TPC of hawthorn leaves collected from 11 different regions of Iran ranged from 19.98 to 82.74 mg GAE g^− 1^ DW, while the TFC varied between 3.34 and 9.90 mg QE g^− 1^ DW. Similarly, Dikici and Köksal [24] reported TPC and TFC values of 161 µg GAE mg^− 1^ extract and 55.6 µg QE mg^− 1^ extract, respectively, for ethanol extracts of hawthorn leaves. Pugna et al. [3] reported a TPC of 199.50 µg GAE mg^− 1^ extract and a TFC of 39.08 µg QE mg^− 1^ extract in hawthorn leaves, while Żurek et al. [25] found that TPC in the leaves of six different hawthorn species ranged from 40.84 to 60.23 mg GAE g^− 1^ DW, with TFC values between 9.45 and 12.77 mg QE g^− 1^ DW. The content and levels of phenolic compounds are influenced by climatic conditions, harvest time, agricultural and environmental factors, as well as post-harvest interventions [18], and TPC is further significantly affected by species, plant organ types, and environmental factors such as harvest time (maturity stage), altitude, light, temperature, and soil nutrient content [24].

The proportion of bound phenolics observed in the present study (approximately 15% of the total phenolic content) was relatively lower compared to values reported in the literature for other plant species and organs. The distribution of bound phenolic compounds in different plant species may vary according to different organs of the plant. For instance, *Terminalia sericea* stems exhibited the highest bound phenolic content (40.89 mg g^− 1^) and bound to total phenolic ratio (73.0%) [26], whereas *Lonicera japonica* stems showed the lowest bound phenolic content (7.93 µmol g^− 1^) [27]. Wang et al. [28] reported that *Rubus idaeus* L. leaves (RDLR) and seed residues (RDSR) contained extractable phenolic contents of 35.42 mg GAE g^− 1^ DM and 6.35 mg GAE g^− 1^ DM, respectively. The bound phenolic contents in the acidic extracts were 31.71 mg GAE/g DM for RDLR and 9.02 mg GAE g^− 1^ DM for RDSR. The contribution of bound phenolics to the total phenolic content (the sum of extractable and bound phenolics) was 47.27% in RDLR and 58.69% in RDSR. Similarly, Durruty et al. [29] reported that free phenolic extracts obtained from sunflower hulls exhibited higher total phenolic (0.26–0.33 g GAE 100 g^-1^ DW) and total flavonoid contents (0.14–0.17 g CE 100 g^-1^ DW) compared to bound phenolic fractions (TPC: 0.18–0.27 g GAE 100 g^-1^ DW; TFC: 0.05–0.09 g CE 100 g^-1^ DW). In contrast, the lower proportion observed in hawthorn leaves may be attributed to differences in cell wall composition and phenolic distribution among species, as well as the predominance of extractable phenolic compounds in leaf tissues. These findings suggest that hawthorn leaves contain relatively more free phenolic compounds than bound forms compared to previously reported plant materials.

### Antioxidant Activity of Free and Bound Phenolic Extracts from Hawthorn Leaves

The antioxidant activities of the extracts are presented in Table 3. The antioxidant activities (via ABTS, DPPH, and FRAP methods) of hawthorn leaf extracts obtained under optimum conditions were evaluated using two extraction techniques: (i) free phenolic extraction by UAE, and (ii) bound phenolic extraction by UAE combined with alkaline.

**Table 3:** Antioxidant activities of free and bound phenolic extracts obtained by UAE under optimum conditions

Antioxidant activity assay	Free phenolic extract (mg TE g⁻¹ DW)	Bound phenolic extract (mg TE g⁻¹ DW)
ABTS	152.28 ± 7.56^A*^	44.91 ± 1.57^B^
DPPH	44.70 ± 2.83^A^	20.81 ± 0.40^B^
FRAP	80.92 ± 8.22^A^	31.86 ± 0.29^B^

*Capital letters in the same line show difference between samples (*p* < 0.05)

Results are given as mean ± standard deviation (*n* = 6)

*ABTS* Cation radical scavenging activity, *DPPH* Free radical scavenging activity, *FRAP* Ferric reducing antioxidant power, *TE* Trolox equivalent

The antioxidant activities of hawthorn leaf extracts obtained under optimum conditions showed that the free phenolic extract exhibited higher activity than the bound phenolic extract across all assays, with ABTS, DPPH, and FRAP. Differences observed among the ABTS, DPPH, and FRAP results may be attributed to the distinct reaction mechanisms of these assays and variations in the composition of the extracts. DPPH and ABTS evaluate radical-scavenging activity, while FRAP measures reducing power. Therefore, differences in phenolic structure and redox properties may lead to variable responses among the assays. In the literature, Martín-García et al. [9] reported DPPH values ranging from 37.68 to 101.3 mg TE g^− 1^ DW, ABTS values from 37.61 to 104.30 mg TE g^− 1^ DW, and FRAP values from 49.62 to 134.68 mg TE g^− 1^ DW under optimized extraction conditions. Similarly, Żurek et al. [25] reported substantially higher ABTS values ranging from 901.04 to 1419.14 mg TE g^− 1^ DW for different hawthorn species. Compared to these findings, the antioxidant activity values obtained in the present study were generally within or slightly above the ranges reported by Martín-García et al. [9], particularly for ABTS, while remaining considerably lower than those reported by Żurek et al. [25]. These differences may be attributed to variations in hawthorn species and environmental growth conditions, differences in polyphenolic composition, and variations in extraction methods and solvent systems. In particular, Żurek et al. [25] emphasised that environmental factors such as ultraviolet (UV) radiation, temperature, humidity, irrigation conditions and soil nutrient availability can significantly affect the phenolic composition of hawthorn and consequently its antioxidant capacity.

In this study, the free phenolic fraction exhibited higher TPC, TFC and antioxidant activity than the bound fraction. This higher antioxidant activity observed in the free phenolic fraction may be attributed to its higher phenolic content compared to the bound fraction, as phenolic compounds are well known to be major contributors to antioxidant activity.

### Individual Phenolic Compound Profiles of Free and Bound Phenolic Extracts from Hawthorn Leaves

Under optimum conditions (i: free phenolic extraction by UAE, and ii: bound phenolic extraction by UAE combined with alkaline extraction), selected individual phenolic compounds (quercetin, quercetin 3-β-D-glucoside, protocatechuic acid, and vitexin) in hawthorn leaf extracts were quantified using LC-MS/MS analysis. The selection of these individual phenolic compounds in this study was based on their reported abundance and biological relevance in hawthorn and hawthorn-derived products [30]. The LC-MS/MS analytical data for the standard compounds quercetin, quercetin 3-β-D-glucoside, protocatechuic acid, and vitexin are presented in Supplementary Material Table S9. The quantities of these compounds detected in the samples are listed in Table 4.

**Table 4:** Individual phenolic compound profiles of extracts obtained by UAE at optimum conditions

Phenolic compound	Free phenolic extract (mg g^− 1^ DW)	Bound phenolic extract (mg g^− 1^ DW)
Quercetin	0.79 ± 0.00^B*^	1.16 ± 0.04^A^
Quercetin-3-β-D-glucoside	13.65 ± 0.23^A^	0.46 ± 0.01^B^
Protocatechuic acid	1.11 ± 0.09^B^	1.47 ± 0.08^A^
Vitexin	6.14 ± 0.03^A^	1.81 ± 0.05^B^

*Capital letters in the same line show difference between samples (*p* < 0.05)

Results are given as mean ± standard deviation (n=6)

The phenolic profiles of free and bound fractions showed clear and statistically significant differences (*p* < 0.05) in the distribution of individual compounds. Quercetin and protocatechuic acid were found at significantly higher levels in the bound phenolic fraction (1.16 and 1.47 mg g^− 1^ DW, respectively) compared to the free fraction (0.79 and 1.11 mg g^− 1^ DW). In contrast, quercetin-3-β-D-glucoside and vitexin were significantly more abundant in the free phenolic fraction (13.65 and 6.14  mg g^− 1^ DW, respectively) than in the bound fraction (0.46 and 1.81 mg g^− 1^ DW). These differences may be attributed to the chemical nature and binding forms of phenolic compounds within the plant matrix. Glycosylated flavonoids such as quercetin-3-β-D-glucoside and vitexin are generally more soluble and readily extractable, which may explain their higher concentration in the free phenolic fraction. In contrast, aglycone forms such as quercetin and simple phenolic acids like protocatechuic acid can be partially bound to cell wall components through ester or ether linkages, leading to their increased presence in the bound phenolic fraction following alkaline hydrolysis.

Several studies have characterized the phenolic profiles of *Crataegus* species. Żurek et al. [25] reported that in leaf extracts of six hawthorn species (*Crataegus* L.), the three most abundant phenolic compounds were procyanidin dimer (41.3%), 4-O-caffeoylquinic acid (37.2%), and quercetin-3-O-galactoside (4.5%). Gao et al. [31] reported vitexin-2-O-rhamnoside as a major compound in *C. pinnatifida* leaves (4.48–5.61 mg g^− 1^), which was also one of the dominant polyphenols in 14 hawthorn leaf species analyzed by Alirezalu et al. [23]; however, this compound was not detected in our study. Ayoub et al. [13] identified bound phenolics such as (+)-catechin, (-)-epicatechin, quercetin, epigallocatechin, myricetin, quercetin pentose, epicatechin gallate, kaempferol hexoside, and quercetin-3-O-glucuronide in the seeds of blackberry, black raspberry, and blueberry. Similarly, in cereals, bound phenolic acids including protocatechuic, 4-hydroxybenzoic, vanillic, *p*-coumaric, and ferulic acids were detected in black rice, with contents of 162.1, 21.2, 27.7, 17.6, and 64.7 µg g^− 1^, respectively [32].

### Antidiabetic Activity of Free and Bound Phenolic Extracts Obtained by UAE from Hawthorn Leaves

The antidiabetic activities of extracts obtained under optimum conditions (i: free phenolic extraction by UAE, and ii: bound phenolic extraction by UAE combined with alkaline extraction) were evaluated using α-amylase and α-glucosidase enzyme inhibition assays (Table 5).

**Table 5:** α-amylase and α-glucosidase inhibition IC_50_ values of hawthorn leaf extracts obtained by UAE and acarbose

Samples	α-amylase (mg mL^− 1^)	α-glucosidase (mg mL^− 1^)
Free phenolic extract	19.18 ± 0.17^b*^	25.70 ± 0.07^b^
Bound phenolic extract	27.56 ± 0.21^a^	26.42 ± 0.13^a^
Acarbose	17.72 ± 0.01^c^	15.07 ± 0.12^c^

*Small letters in the same column show difference between samples (p < 0.05)

Results are given as mean ± standard deviation (n=6)

The α-amylase and α-glucosidase inhibitory activities of hawthorn leaf extracts were determined, yielding IC_50_ values of 19.19–27.56 mg mL^− 1^ and 25.70–26.42 mg mL^− 1^, respectively. In contrast, lower IC_50_ values have been reported in the literature. For example, Skaf [33] reported IC_50_ values of 0.13–0.15 mg mL^−1^and 0.10–0.12 mg mL^− 1^ for α-amylase in *Crataegus monogyna* and *Crataegus azarolus* leaf extracts, respectively. Similarly, Żurek et al. [25] reported IC_50_ values ranging from 0.80 to 1.37 mg mL^− 1^ for α-amylase and from 1.27 to 2.14 mg mL^− 1^ for α-glucosidase in different hawthorn species. Compared to these findings, the IC_50_ values obtained in the present study are considerably higher, indicating the extracts’ lower inhibitory potency. Furthermore, when compared with the reference inhibitor acarbose, the extracts exhibited lower inhibitory activity. These differences may be attributed to variations in hawthorn species and environmental growth conditions, as well as differences in phenolic composition, the distribution of free and bound phenolic fractions, extraction methods, solvent systems and assay conditions. For example, Haideri et al. [34] demonstrated that the extraction method significantly affects antidiabetic activity, with different techniques producing extracts with varying α-amylase and α-glucosidase inhibitory potentials.

In this study, the lower IC_50_ values observed for the free phenolic fraction indicate a stronger enzyme inhibitory activity, which may be attributed to its higher content and greater accessibility of phenolic compounds, known to play a key role in α-amylase and α-glucosidase inhibition.

In diabetes management, the control of postprandial blood glucose levels is commonly achieved through dietary strategies and pharmacological approaches, including enzyme inhibitors targeting carbohydrate digestion such as α-amylase and α-glucosidase inhibitors (e.g., acarbose). These inhibitors act by delaying the hydrolysis of complex carbohydrates, thereby reducing glucose absorption. However, gastrointestinal side effects associated with synthetic inhibitors have increased interest in plant-derived compounds, particularly those used in traditional medicine [35]. In this context, phenolic compounds have been reported to exhibit inhibitory effects on α-amylase and α-glucosidase in vitro, suggesting their potential role in modulating carbohydrate digestion. In addition, their antioxidant properties may contribute to the reduction of oxidative stress associated with metabolic disorders [36]. Nevertheless, it should be noted that these effects are based on in vitro assays and do not directly reflect in vivo antidiabetic efficacy.

## Conclusion

This study demonstrated that hawthorn (*Crataegus azarolus*) leaves are a rich source of phenolic compounds, predominantly present in the free fraction. Under optimized conditions, free phenolic content reached 78.61 mg GAE g^-1^ DW, while bound phenolic content reached 13.56 mg GAE g^− 1^ DW, corresponding to approximately 85% and 15% of total phenolics, respectively. Extracts obtained under optimized conditions exhibited antioxidant activity and in vitro α-amylase and α-glucosidase inhibitory effects. These findings highlight the potential of hawthorn leaves as a source of bioactive compounds and confirm the effectiveness of UAE for phenolic recovery. For bound phenolics, the combination of alkaline hydrolysis and UAE proved to be an efficient strategy to release cell wall–associated compounds, thereby improving their extractability compared to conventional approaches. Overall, this study provides new insights into the phenolic profile of hawthorn leaves and supports their potential as an underutilized plant material for value-added applications. Future studies should focus on improving extraction efficiency at larger scales, investigating the stability and bioaccessibility of hawthorn leaf phenolics, and evaluating their incorporation into functional food systems.

## Supplementary Information

Below is the link to the electronic supplementary material.

Supplementary Material 1

## Data Availability

Data will be made available on request.
