# Supplementary material for: Ultrasound-Assisted Extraction of Free and Bound Phenolics from Hawthorn (Crataegus azarolus) Leaves and their Antioxidant and Antidiabetic Activities
Source: Plant Foods Hum Nutr. 2026 May 16;81(2):64. doi: 10.1007/s11130-026-01505-0 (PMC13179261; doi:10.1007/s11130-026-01505-0)
Supplement: Supplementary file 1 — Supplementary Material 1 [file 11130_2026_1505_MOESM1_ESM.docx]

**Supplementary Materials**

**Ultrasound-Assisted Extraction of Free and Bound Phenolics from Hawthorn**

**(*Crataegus azarolus*) Leaves and Their Antioxidant and Antidiabetic Activities**

Esra ESİN^1*^, Semra TOPUZ TÜRKER^1^, Mustafa BAYRAM^1^, Cemal KAYA^1^

**^1^** Department of Food Engineering, Tokat Gaziosmanpasa University, 60250 Tokat, Türkiye

***Corresponding author:** Esra ESİN

Tokat Gaziosmanpasa University, Faculty of Engineering and Architecture,

Department of Food Engineering, 60250, Tokat, Türkiye

E-mail**:** esinyasemin@yahoo.com

Orcid ID: <https://orcid.org/0000-0003-0470-0015>

**Material and Methods**

**Material**

**Plant Material**

The hawthorn (*Crataegus azarolus)* leaves used as raw material in this study were collected in September 2022. The collection was planned to coincide with the hawthorn fruit harvest period. Leaves were gathered from Soğukpınar area in Çayır Village, Zile district of Tokat province, located at an altitude of 1860 meters. The collected leaves were dried at room temperature before the extraction process. After drying, the samples were ground into powder using a grinder and passed through a sieve with a pore size of 0.075 mm^2^. The prepared hawthorn leaf powder was stored in glass jars at room temperature and in dark conditions until extraction. The jars were sealed tightly and wrapped with parafilm to protect against external factors.

**Chemical Materials and Equipments**

Analytical-grade reagents used in this study included gallic acid (C₇H₆O₅, Sigma, Germany), ethanol (C₂H₅OH, Tekkim, Türkiye), ethyl acetate (≥99.5%, Tekkim, Türkiye), sodium nitrite (NaNO₂, Merck, Germany), sodium hydroxide (NaOH, Merck, Germany), aluminum chloride (AlCl₃, Merck, Germany), sodium carbonate (Na₂CO₃, Isolab, Germany), potassium persulfate (K₂S₂O₈, Merck, Germany), Folin-Ciocalteu reagent (Carlo Erba, France), Trolox ((±)-6-hydroxy-2,5,7,8-tetramethylchroman-2-carboxylic acid, Sigma, Germany), ABTS (2,2′-azino-bis(3-ethylbenzothiazoline-6-sulfonic acid) diammonium salt, Sigma, Germany), DPPH (2,2-diphenyl-1-picrylhydrazyl, Sigma, Germany), quercetin (BLD Pharm, China), hydrochloric acid (HCl, Sigma, Germany), sodium acetate (CH₃COONa, Merck, Germany), iron(III) chloride (FeCl₃, Sigma, Germany), TPTZ (2,4,6-tri(2-pyridyl)-1,3,5-triazine, Tokyo Chemical Industry, Japan), α-amylase (*Aspergillus oryzae*, 30 U/mg, Sigma, Germany), acarbose (BLD Pharm, China), and α-glucosidase (*Saccharomyces cerevisiae*, ≥10 U/mg, Sigma, Germany).

The equipment used included a UV-VIS spectrophotometer (PG Instruments, T80+, UK),
LC-MS/MS device (Shimadzu, Japan), ultrapure water system (Merck Millipore, Germany), analytical balance (Radwag AS 220 R2, Poland), centrifuge (Nüve, Türkiye), grinder (Sinbo SCM 2934, Türkiye), vortex mixer (Velp Scientifica, Italy), water bath (Memmert, Germany), magnetic stirrer with heating function (Biosan MSH 300, Latvia), and ultrasonic bath (Elma, Germany).

**Method**

**Optimization of Free Phenolic Compound Extraction Using UAE Technique**

Free phenolic compounds were extracted from hawthorn leaf powder using UAE with a modified method based on Irakli et al. [1]. Extraction conditions were optimized using Design-Expert software (v.7.0, Stat-Ease Inc., USA) with Response Surface Methodology (RSM) based on a Box–Behnken design of three variables at three levels. Hawthorn leaf powder (400 mg) was extracted in an ultrasonic bath (37 kHz, continious mode) using 10 mL ethanol (EtOH)/water mixtures at concentrations ranging from 10% to 90%. Extraction times varied between 5 and 90 minutes, and temperatures ranged from 30 to 70 ⁰C. The extracts were centrifuged at 6000 rpm for 5 minutes, filtered through coarse filter paper, and the resulting filtrates were used for analysis. The range of independent variables was determined based on preliminary experiments and literature review. Extraction conditions were optimized for EtOH concentration, extraction time, and temperature. The optimal conditions were those that produced the highest total phenolic content (TPC) and total flavonoid content (TFC). The experimental design used in this study is presented in Table S1. The effects of the extraction parameters on TPC and TFC were analyzed, and the extraction process was optimized using the desirability function approach to obtain the highest yield. Significant terms in the mathematical models for extraction yield were identified by analysis of variance (ANOVA). Following the optimization based solely on TPC and TFC, the antioxidant (ABTS, DPPH, and FRAP) and antidiabetic (α-amylase and α-glucosidase inhibition) activities of the phenolic extracts obtained under optimum conditions were evaluated, and selected individual phenolic compounds were further characterized by LC-MS/MS.

**Table S1.** Experimental design for free phenolic extraction using UAE

| **Trial no** | **Extraction time (min)  (X_1_)** | **Temperature (°C)**  **(X_2_)** | **EtOH concentration (%)**  **(X_3_)** |
| --- | --- | --- | --- |
| 1 | 47.50 | 50.00 | 50.00 |
| 2 | 47.50 | 50.00 | 50.00 |
| 3 | 47.50 | 30.00 | 90.00 |
| 4 | 90.00 | 30.00 | 50.00 |
| 5 | 90.00 | 50.00 | 10.00 |
| 6 | 47.50 | 50.00 | 50.00 |
| 7 | 90.00 | 50.00 | 90.00 |
| 8 | 5.00 | 70.00 | 50.00 |
| 9 | 47.50 | 70.00 | 10.00 |
| 10 | 47.50 | 30.00 | 10.00 |
| 11 | 47.50 | 50.00 | 50.00 |
| 12 | 5.00 | 30.00 | 50.00 |
| 13 | 47.50 | 70.00 | 90.00 |
| 14 | 90.00 | 70.00 | 50.00 |
| 15 | 47.50 | 50.00 | 50.00 |
| 16 | 5.00 | 50.00 | 90.00 |
| 17 | 5.00 | 50.00 | 10.00 |

**Optimization of Bound Phenolic Compound Extraction Using UAE Technique**

Extraction of bound phenolic compounds from hawthorn leaf powder using alkaline treatment combined with UAE was performed with a modified method based on Irakli et al. [1]. To optimize the extraction of bound phenolic compounds, free phenolics must first be removed. Therefore, 400 mg of powder leaf samples were extracted under the optimal conditions previously determined for free phenolic compound extraction. The supernatant was separated, and the remaining residue was used for the subsequent alkaline UAE hydrolysis step. The residue was suspended in 10 mL of NaOH at varying concentrations (1 to 4 M), and hydrolysis was carried out in an ultrasonic water bath (37 kHz, continuous mode) at different times (5 to 90 minutes) and temperatures (30 to 70 °C). The mixture was then acidified to pH 2 with concentrated HCl, centrifuged at 6000 rpm for 5 minutes, and filtered through coarse filter paper. The obtained supernatant was subjected twice to liquid-liquid extraction using 25 mL of ethyl acetate. The ethyl acetate phase was separated using a separatory funnel and evaporated at 40 °C under reduced pressure using a rotary evaporator. The extract in the flask was reconstituted in 10 mL of EtOH/water mixture (50:50, v/v), and the resulting filtrates were used for analysis. The ranges for independent variables were selected based on preliminary tests and literature review. Conditions that yielded the highest TPC and TFC were chosen as optimal. The experimental design used in this study is shown in Table S2. The effects of process variables specific to the extraction method on TPC and TFC were examined, and the extraction process was optimized using the desirability function approach to obtain extracts with the highest values. Significant terms in the mathematical models for extraction yield were identified by analysis of variance. Following the optimization based solely on TPC and TFC, the antioxidant (ABTS, DPPH, and FRAP) and antidiabetic (α-amylase and α-glucosidase inhibition) activities of the phenolic extracts obtained under optimum conditions were evaluated, and selected individual phenolic compounds were further characterized by LC-MS/MS.

**Table S2.** Experimental design for the extraction of bound phenolics using UAE

| **Trial no** | **NaOH concentration (M)**  **(X_1_)** | **Hydrolysis time (min)  (X_2_)** | **Temperature (°C)**  **(X_3_)** |
| --- | --- | --- | --- |
| 1 | 1.00 | 47.50 | 30.00 |
| 2 | 1.00 | 90.00 | 50.00 |
| 3 | 2.50 | 47.50 | 50.00 |
| 4 | 2.50 | 5.00 | 30.00 |
| 5 | 2.50 | 5.00 | 70.00 |
| 6 | 2.50 | 47.50 | 50.00 |
| 7 | 2.50 | 90.00 | 30.00 |
| 8 | 4.00 | 5.00 | 50.00 |
| 9 | 4.00 | 47.50 | 30.00 |
| 10 | 4.00 | 90.00 | 50.00 |
| 11 | 1.00 | 5.00 | 50.00 |
| 12 | 2.50 | 90.00 | 70.00 |
| 13 | 2.50 | 47.50 | 50.00 |
| 14 | 1.00 | 47.50 | 70.00 |
| 15 | 4.00 | 47.50 | 70.00 |
| 16 | 2.50 | 47.50 | 50.00 |
| 17 | 2.50 | 47.50 | 50.00 |

**Determination of TPC**

TPC values were determined using the Folin-Ciocalteu (FC) method. A 100 μL aliquot of the extract solution was mixed with 200 μL of FC reagent and 2 mL of distilled water, then left at room temperature for 3 minutes. Afterwards, 1 mL of 20% sodium carbonate (Na_2_CO_3_) solution was added, and the mixture was vortexed. The solution was incubated at room temperature for
1 hour. Following incubation, the absorbance of the mixture was measured at 765 nm using a spectrophotometer. TPC values were calculated using a calibration curve prepared with gallic acid standards and expressed as mg gallic acid equivalents (GAE) g^-1^ dry weight (DW)[2].

**Determination of TFC**

TFC values were determined using a modified method based on Gaafar and Salama [3]. To 500 μL of extract, 2 mL of distilled water and 150 μL of 5% sodium nitrite (NaNO_2_) solution were added. After standing at room temperature for 5 minutes, 150 μL of 10% aluminum chloride (AlCl_3_) solution was added. The mixture was allowed to react for 5 minutes, then 1 mL of 1 M sodium hydroxide (NaOH) and 1.2 mL of distilled water were added, followed by vortex mixing. The absorbance of the mixture was measured at 415 nm using a spectrophotometer. TFC values were calculated using a calibration curve prepared with quercetin standards and expressed as mg quercetin equivalents (QE) g^-1^ DW.

**Cation Radical Scavenging Activity (ABTS^●+^)**

The antioxidant activity of the samples was determined using the spectrophotometric method developed by Re et al. [4]. For this purpose, 7 mM ABTS and 2.45 mM potassium persulfate (K_2_S_2_O_8_) solutions were prepared. To prepare the ABTS radical stock solution, ABTS and K_2_S_2_O_8_ solutions were mixed in a 1:1 ratio and incubated at room temperature in the dark for 16 hours. The working ABTS radical solution was prepared by diluting 1 mL of the stock solution with ethanol to obtain an absorbance of 0.700 (±0.02) at 734 nm. Then, 40 μL of each sample was mixed with 4 mL of the ABTS radical solution and incubated at room temperature in the dark for 6 minutes to allow the reaction to proceed. Absorbance was measured at 734 nm using a spectrophotometer. The cation radical scavenging activity of the samples was calculated using a calibration curve prepared with Trolox standard and expressed as mg Trolox equivalents g^-1^ DW.

**Free Radical Scavenging Activity (DPPH^●^)**

The free radical scavenging activity of the samples against DPPH was determined according to the method of Blasi et al. [5]. First, a 0.06 mM DPPH solution was prepared and stored in the dark at 4 °C for 1 hour. Then, 100 μL of each sample was mixed with 3.9 mL of the DPPH solution and vortexed. The mixture was incubated in the dark at room temperature for 30 minutes. After incubation, the absorbance was measured at 517 nm using a spectrophotometer. The free radical scavenging activity was calculated using a calibration curve prepared with Trolox standards and expressed as mg Trolox equivalents g^-1^ DW.

**Ferric Reducing Power Activity (FRAP)**

The antioxidant activity of the samples was determined using the FRAP method according to Benzie and Strain [6]. The working solution was prepared by mixing acetate buffer (30 mM, pH 3.6), FeCl_3_ (20 mM), and TPTZ solution (10 mM) in a ratio of 10:1:1. A volume of 2.9 mL of the FRAP working solution was added to 100 μL of the sample and vortexed. The mixture was incubated at room temperature for 30 minutes, after which the absorbance was measured at 593 nm using a spectrophotometer. FRAP values were calculated using a calibration curve prepared with Trolox standards and expressed as mg Trolox equivalents g^-1^ DW.

**Individual phenolic compound profile analysis by LC-MS/MS**

Determination of individual phenolics in hawthorn leaf extracts obtained at UAE was carried out by Topuz Türker & Bayram [7] method using LC–MS/MS (LC-MS 8050, Shimadzu). Quantitative analysis of quercetin, quercetin 3-β-D-glucoside, protocatechuic acid, and vitexin in the samples were performed using an LC-MS/MS system. Stock solutions of the standards were prepared in methanol and stored at -18 °C. Samples were diluted by adding ethanol prior to analysis. A 5 μL aliquot of the prepared samples was filtered through a 0.22 μm membrane filter (Millex-HV). The filtrates were placed into the sample vials of the LC-MS/MS device. For this purpose, the extracts were filtered through a membrane filter with a pore diameter of 0.45µm and then injected into the LC–MS/MS device. Chromatographic separation was achieved on a C18 column (150 × 2.0 mm, 3 µm) at 40 °C using ammonium formate (10 mmol/L) in water (A) and methanol (B) at a flow rate of 0.40 mL/min. Mass spectrometric detection was conducted using electrospray ionization in positive mode (ESI⁺). Detailed LC–MS/MS operating conditions are provided in the Table S3 and S4. Results are expressed in mg per gram of dry sample.

**Table S3.** Operating conditions of LC-MS/MS analysis

| **LC conditions (Nexera X2)** | | **MS conditions (LCMS-8050)** | |
| --- | --- | --- | --- |
| **Column** | Inertsil (ODS-4), C18 Column (150 x 2.00 mm, 3 μm) | **Ionization mode** | ESI (Positive) |
| **Oven temperature (°C)** | 40 ℃ | **Desolvation line temperature (°C)** | 250 ⁰C |
| **Mobile phase A** | 10 mmol/L Ammonium formate/deionized water | **Interface temperature** | 300 ⁰C |
| **Mobile phase B** | Metanol | **Block heater temperature** | 400 ⁰C |
| **Flow rate** | 0.40 mL/min | **Nebulizer gas flow** | 3 L/min |
| **Injection volume** | 5 μL | **Drying gas flow** | 10 L/ min |
| **Rinse solution** | R0: %50 methanol | **Heating gas flow** | 15 L/ min |
|  |  | **Dwell time** | 1-33 ms |

**Table S4.** Gradient system solvent composition for LC-MS/MS

| **Time (min)** | **Mobile phase A (%)** | **Mobile phase B (%)** |
| --- | --- | --- |
| 0.00 | 80 | 20 |
| 3.00 | 30 | 70 |
| 10.30 | 5 | 95 |
| 10.51-14.00 | 80 | 20 |

**α-amylase Enzyme Inhibition Activity**

The α-amylase inhibition assay was conducted based on previously reported methods [8, 9] α-amylase dissolved in phosphate buffer (0.02 M, pH 6.9) was mixed with extracts diluted at different concentrations in separate tubes and incubated at 37 °C for 10 minutes. Then, 1% starch solution was added as substrate, and the mixture was incubated at 37 °C for 15 minutes. The reaction was stopped by adding dinitro salicylic acid reagent, and the tubes were heated in boiling water for 10 minutes. After cooling, the mixture was diluted with distilled water. Absorbance was measured at 540 nm. The same procedure was performed using acarbose as the standard inhibitor. The α-amylase inhibition activity of the extracts was calculated graphically and expressed as IC_50_ in mg mL^-1^.

Inhibition (%) = [(A_0_ – A_i_) / A_0_] × 100

where A_0_ is the average absorbance without the sample and A_i_ is the average absorbance with the sample at different concentrations.

**α-Glucosidase Enzyme Inhibition Activity**

The α-glucosidase inhibition assay was performed based on previously reported methods [8, 9]. For the determination of α-glucosidase inhibition activity, α-glucosidase dissolved in phosphate buffer (0.1 M, pH 6.8) was mixed with extracts diluted at various concentrations in separate tubes and incubated at 37 °C for 10 minutes. Then,
4-nitrophenyl α-D-glucopyranoside substrate was added, and the tubes were incubated at 37 °C for 15 minutes. The reaction was stopped by adding Na_2_CO_3_, and absorbance was recorded at 405 nm. The same procedure was repeated using acarbose as the standard inhibitor. The
α-glucosidase inhibition activity of the extracts was calculated graphically and expressed as IC_50_ in mg mL^-1^.

Inhibition (%) = [(A_0_ – A_i_) / A_0_] × 100

where A_0_ is the average absorbance without the sample and A_i_ is the average absorbance with the sample at different concentrations.

**Statistical Analysis**

Statistical analyses were performed using SPSS 22.0 (IBM, USA) and Design Expert 7.0 (Stat-Ease Inc., USA) software packages. All experiments were conducted with two independent replicates and three parallel measurements**.** The t-test and calculation of Pearson correlation coefficients between samples were conducted using SPSS 22.0 to determine differences between predicted values and experimental data. Regression analysis, statistical evaluations, isohips contour plots, response surface graphs, and optimization related to the effects of process variables on TPC and TFC were performed using Design Expert 7.0.

**Table S5.** Effects of extraction parameters on TPC and TFC in free phenolic extraction by UAE

| **Source of variation** | **Degrees of freedom** | **Sum of squares** | | **Mean square** | | **F value** | | **P value** | |
| --- | --- | --- | --- | --- | --- | --- | --- | --- | --- |
|  |  | **TPC** | **TFC** | **TPC** | **TFC** | **TPC** | **TFC** | **TPC** | **TFC** |
| **Model** | 9 | 3924.02 | 2592.44 | 436.00 | 288.05 | 193.16 | 102.51 | <0.0001 | <0.0001 |
| **X_1_** | 1 | 116.92 | 136.36 | 116.92 | 136.36 | 51.80 | 48.53 | 0.0002 | 0.0002 |
| **X_2_** | 1 | 232.70 | 86.51 | 232.70 | 86.51 | 103.09 | 30.79 | <0.0001 | 0.0009 |
| **X_3_** | 1 | 817.17 | 1017.05 | 817.17 | 1017.05 | 362.03 | 361.93 | <0.0001 | <0.0001 |
| **X_1_X_2_** | 1 | 97.31 | 24.19 | 97.31 | 24.19 | 43.11 | 8.61 | 0.0003 | 0.0219 |
| **X_1_X_3_** | 1 | 0.20 | 2.61 | 0.20 | 2.61 | 0.089 | 0.93 | 0.7743 | 0.3673 |
| **X_2_X_3_** | 1 | 47.55 | 0.28 | 47.55 | 0.28 | 21.07 | 0.10 | 0.0025 | 0.7595 |
| **X_1_^2^** | 1 | 37.66 | 10.08 | 37.66 | 10.08 | 16.68 | 3.59 | 0.0047 | 0.1000 |
| **X_2_^2^** | 1 | 28.01 | 0.46 | 28.01 | 0.46 | 12.41 | 0.16 | 0.0097 | 0.6993 |
| **X_3_^2^** | 1 | 2464.58 | 1291.53 | 2464.58 | 1291.53 | 1091.88 | 459.61 | <0.0001 | <0.0001 |
| **Residual** | 7 | 15.80 | 19.67 | 2.26 | 2.81 |  |  |  |  |
| **Lack of fit** | 3 | 6.00 | 5.56 | 2.00 | 1.85 | 0.82 | 0.53 | 0.5485 | 0.6880 |
| **Pure error** | 4 | 9.80 | 14.11 | 2.45 | 3.53 |  |  |  |  |
| **Total** | 16 | 3939.82 | 2612.11 |  |  |  |  |  |  |
|  | **R^2^** | **Adj- R^2^** | | **Adequate Prediction** | | **PRESS** | | **C.V (%)** | |
| **TPC** | 0.99 | 0.99 | | 47.23 | | 111.28 | | 2.70 | |
| **TFC** | 0.99 | 0.98 | | 32.40 | | 111.02 | | 3.68 | |

X_1_: Extraction time (min), X_2_: Temperature (°C), X_3_: Ethanol concentration (%), TPC: Total phenolic compound, TFC: Total flavonoid compound.


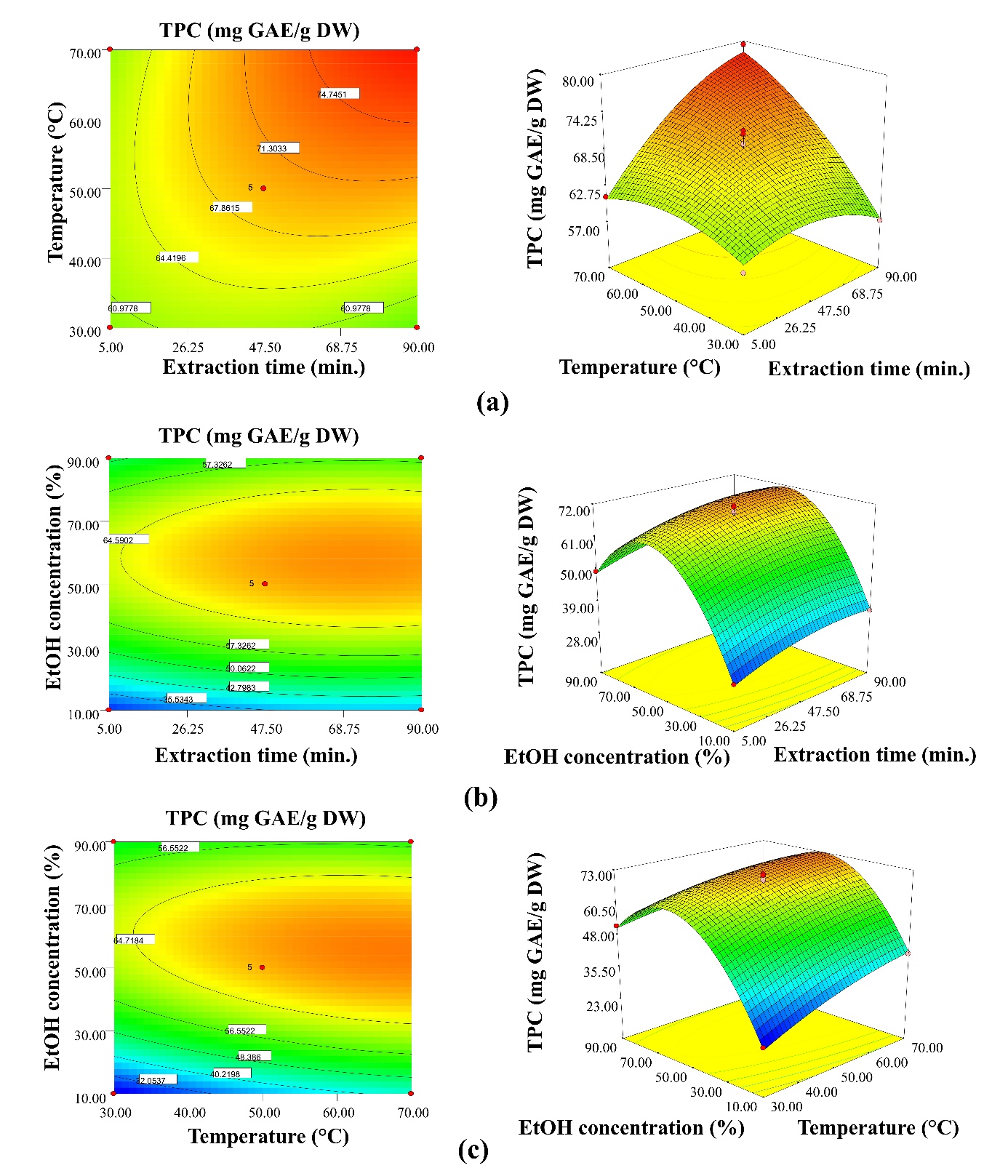


**Figure S1**. Response surface plots showing the effects **(a)** extraction time and temperature, **(b)** extraction time and EtOH concentration, and **(c)** temperature and EtOH concentration on TPC values obtained from hawthorn leaves by UAE for free phenolic extraction


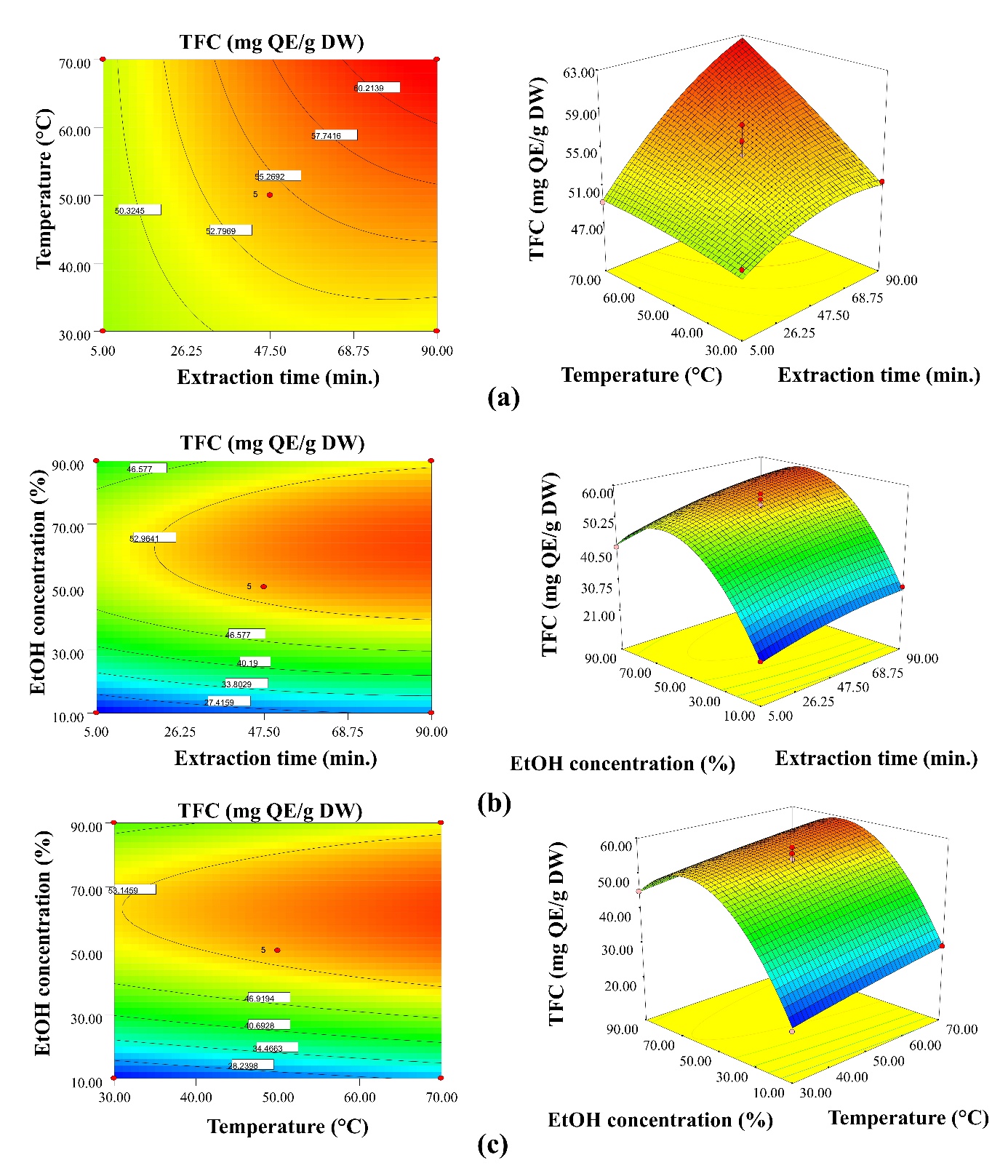


**Figure S2**. Response surface plots showing the effects of **(a)** extraction time and temperature, **(b)** extraction time and EtOH concentration, and **(c)** temperature and EtOH concentration on TFC values obtained from hawthorn leaves by UAE for free phenolic extraction

| 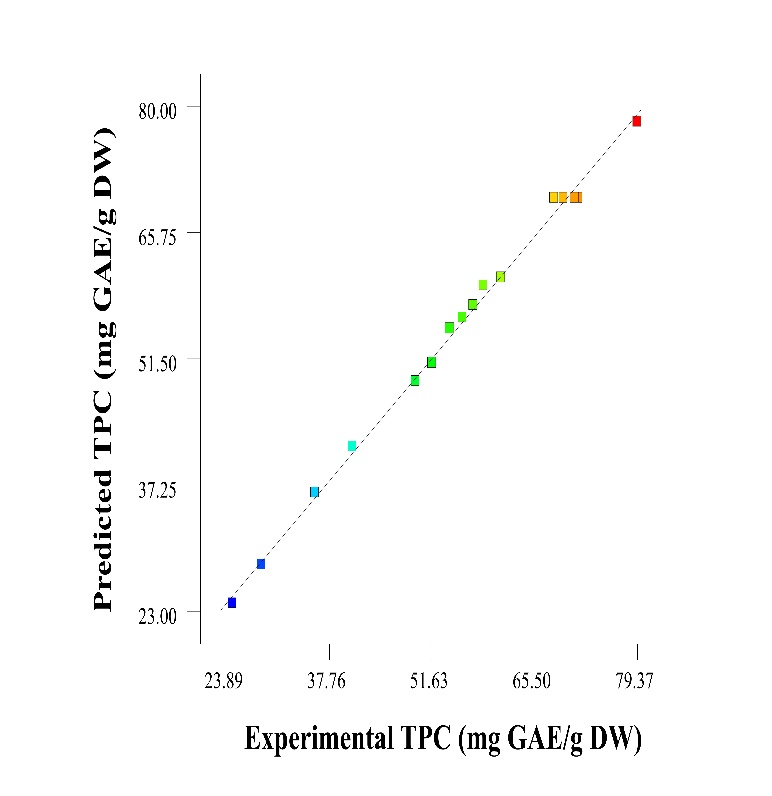 | 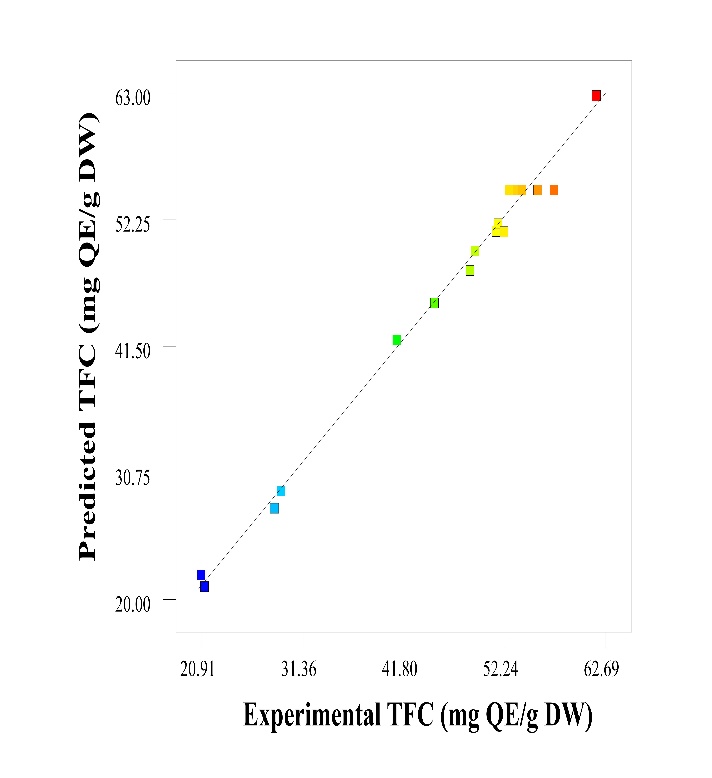 |
| --- | --- |
| **(a)** | **(b)** |

**Figure S3**. Relationship between experimental and predicted values for **(a)** TPC and **(b)** total TFC in free phenolic extraction using UAE

In Figure S3(a) and Figure S3(b), the experimental data for TPC and TFC are compared with the predicted values from the polynomial models, showing that the data points align closely along the 45° line. This strong agreement indicates a high predictive accuracy of the developed models and confirms the suitability and reliability of the polynomial models in describing the extraction behavior of bound phenolic compounds from hawthorn leaves (R^2^ = 0.99 for TPC and R^2^ = 0.99 for TFC).

**Table S6.** Optimum extraction conditions for free phenolic extraction by UAE

| **No** | **Extraction time (min)** | **Temperature (°C)** | **EtOH concentration (M)** | **TPC (mg GAE/g DW)** | **TFC (mg QE/g DW)** | **Desirability** |
| --- | --- | --- | --- | --- | --- | --- |
| 1 | 90.00 | 70.00 | 55.33 | 78.61 | 63.95 | 0.99 |
| 2 | 90.00 | 69.97 | 56.07 | 78.60 | 64.07 | 0.99 |
| 3 | 90.00 | 69.44 | 54.88 | 78.48 | 63.73 | 0.99 |
| 4 | 87.76 | 70.00 | 56.67 | 78.43 | 63.97 | 0.99 |

**Table S7.** Effects of extraction parameters on TPC and TFC in bound phenolic extraction by UAE

| **Source of variation** | **Degrees of freedom** | **Sum of squares** | | **Mean square** | | **F value** | | **P value** | |
| --- | --- | --- | --- | --- | --- | --- | --- | --- | --- |
|  |  | **TPC** | **TFC** | **TPC** | **TFC** | **TPC** | **TFC** | **TPC** | **TFC** |
| **Model** | 9 | 254.79 | 130.89 | 28.31 | 14.54 | 52.26 | 43.02 | <0.0001 | <0.0001 |
| **X_1_** | 1 | 45.77 | 25.58 | 45.77 | 25.58 | 84.49 | 75.66 | <0.0001 | <0.0001 |
| **X_2_** | 1 | 121.18 | 67.01 | 121.18 | 67.01 | 223.68 | 198.22 | <0.0001 | <0.0001 |
| **X_3_** | 1 | 3.55 | 1.25 | 3.55 | 1.25 | 6.55 | 3.69 | 0.0376 | 0.0964 |
| **X_1_X_2_** | 1 | 0.27 | 0.00004 | 0.27 | 0.00004 | 0.50 | 0.00012 | 0.5031 | 0.9915 |
| **X_1_X_3_** | 1 | 6.26 | 5.13 | 6.26 | 5.13 | 11.55 | 15.17 | 0.0115 | 0.0059 |
| **X_2_X_3_** | 1 | 0.74 | 0.0015 | 0.74 | 0.0015 | 1.37 | 0.0044 | 0.2805 | 0.9491 |
| **X_1_^2^** | 1 | 4.04 | 1.05 | 4.04 | 1.05 | 7.46 | 3.10 | 0.0292 | 0.1215 |
| **X_2_^2^** | 1 | 51.80 | 20.21 | 51.80 | 20.21 | 95.61 | 59.77 | <0.0001 | 0.0001 |
| **X_3_^2^** | 1 | 15.15 | 8.21 | 15.15 | 8.21 | 27.96 | 24.29 | 0.0011 | 0.0017 |
| **Residual** | 7 | 3.79 | 2.37 | 0.54 | 0.34 |  |  |  |  |
| **Lack of fit** | 3 | 2.20 | 1.89 | 0.73 | 0.63 | 1.85 | 5.24 | 0.2792 | 0.0717 |
| **Pure error** | 4 | 1.59 | 0.48 | 0.40 | 0.12 |  |  |  |  |
| **Total** | 16 | 258.58 | 133.25 |  |  |  |  |  |  |
|  |  |  |  |  |  |  |  |  |  |
|  | **R^2^** | **Adj- R^2^** | | **Adequate Prediction** | | **PRESS** | | **C.V (%)** | |
| **TPC** | 0.99 | 0.97 | | 22.26 | | 37.72 | | 8.75 | |
| **TFC** | 0.98 | 0.96 | | 20.99 | | 30.93 | | 9.63 | |

X_1_: NaOH concentration (M), X_2_: Hydrolysis time (min), X_3_: Temperature (°C), TPC: Total phenolic compound, TFC: Total flavonoid compound.


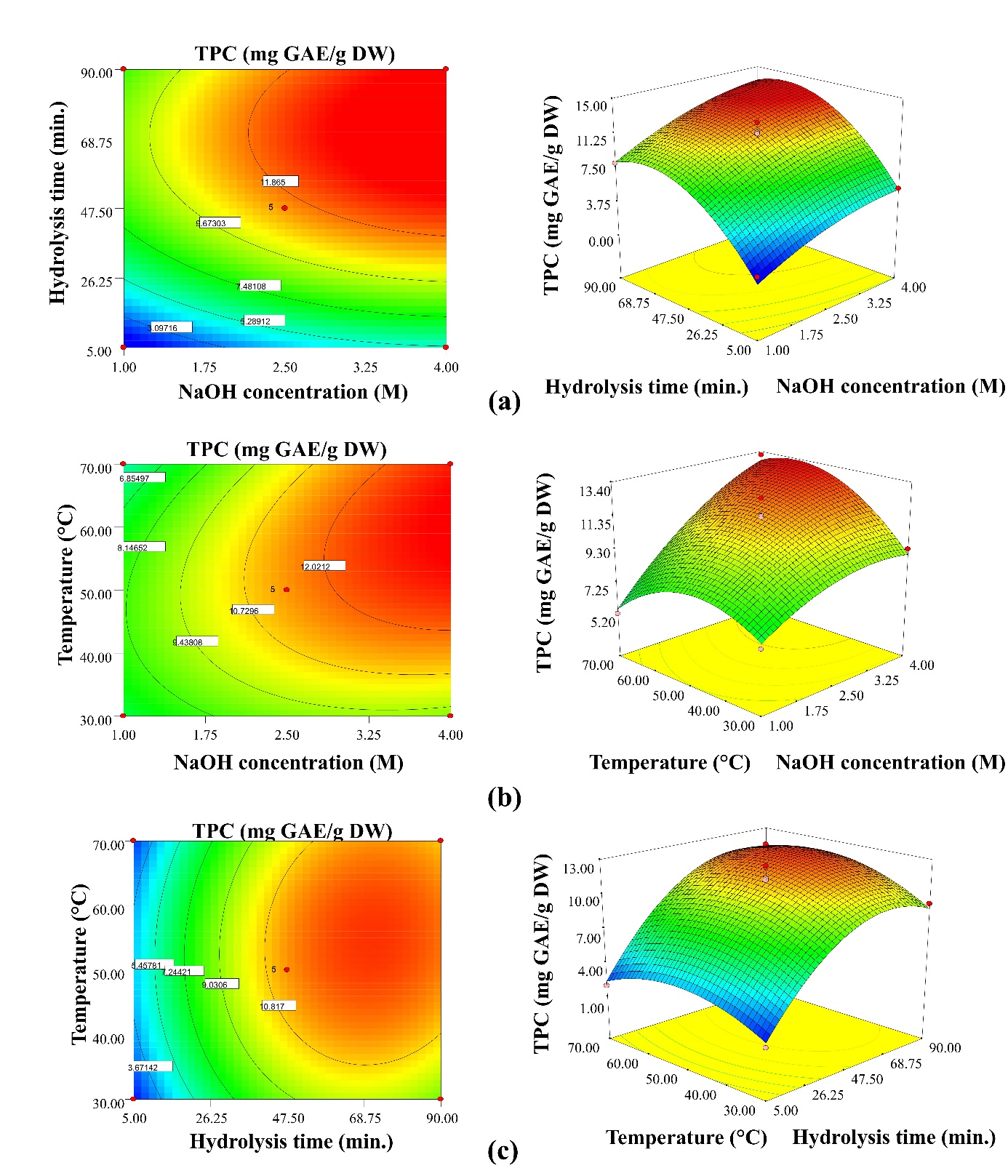


**Figure S4**. Response surface plots showing the effects of **(a)** hydrolysis time and NaOH concentration, **(b)** temperature and NaOH concentration, and **(c)** temperature and hydrolysis time on the TPC values obtained from hawthorn leaves by UAE of bound phenolics


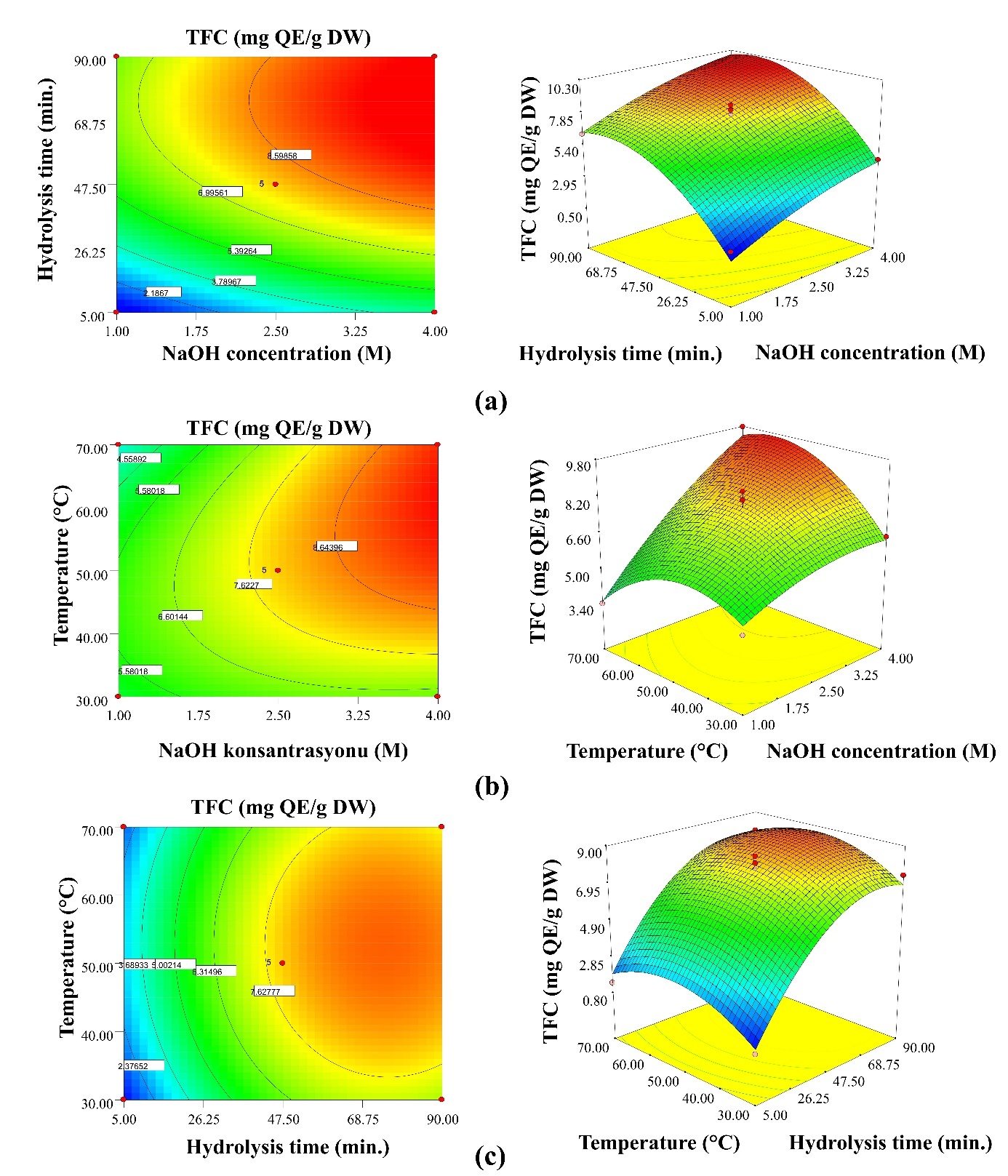


**Figure S5**. Response surface plots showing the effects of **(a)** hydrolysis time and NaOH concentration, **(b)** temperature and NaOH concentration, and **(c)** temperature and hydrolysis time on the TFC values obtained from hawthorn leaves by UAE of bound phenolics

| 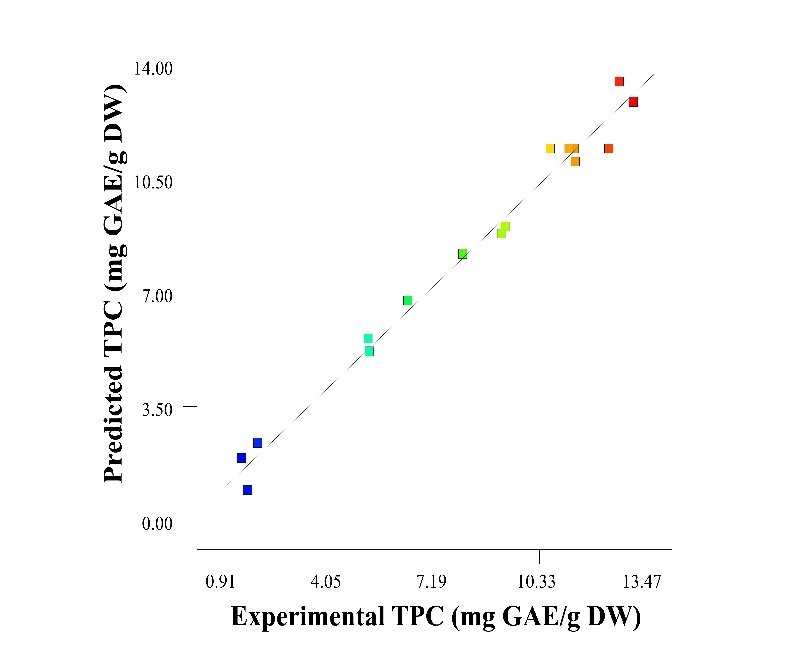 | 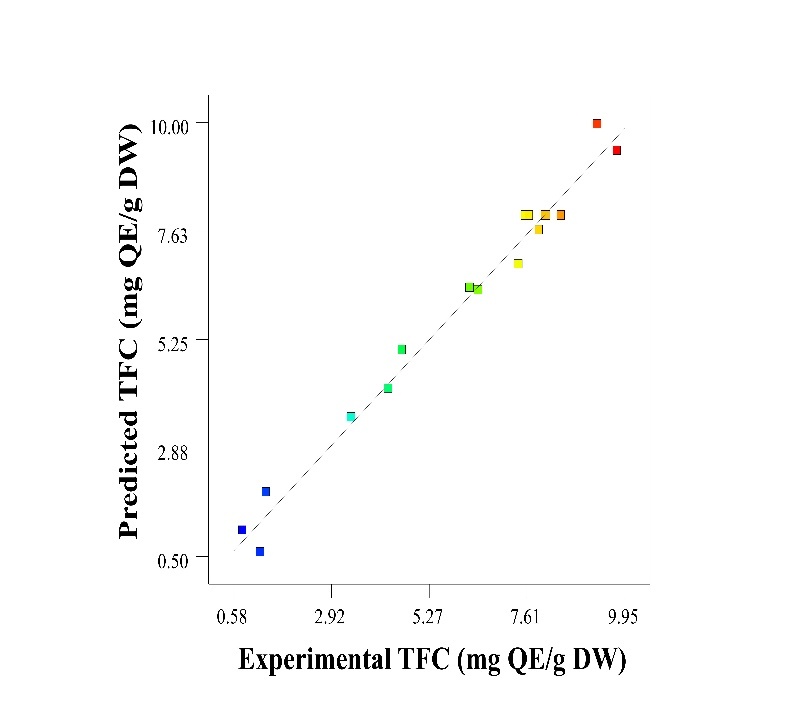 |
| --- | --- |
| **(a)** | **(b)** |

**Figure S6**. Relationship between experimental and predicted values obtained for **(a)** TPC and **(b)** TFC during bound phenolic extraction from hawthorn leaves using UAE

In Figure S6(a) and Figure S6(b), the experimental data for TPC and TFC are compared with the predicted values from the polynomial models, showing that the data points align closely along the 45° line. This strong agreement indicates a high predictive accuracy of the developed models and confirms the suitability and reliability of the polynomial models in describing the extraction behavior of bound phenolic compounds from hawthorn leaves (R² = 0.98 for TPC and R² = 0.98 for TFC).

**Table S8**. Optimum extraction conditions for bound phenolic extraction by UAE

| **No** | **NaOH concentration (M)** | **Time (min)** | **Temperature (°C)** | **TPC (mg GAE/g DW)** | **TFC (mg QE/g DW)** | **Desirability** |
| --- | --- | --- | --- | --- | --- | --- |
| 1 | 3.87 | 83.35 | 48.24 | 13.56 | 9.92 | 1.00 |
| 2 | 3.59 | 76.72 | 49.53 | 13.74 | 9.92 | 1.00 |
| 3 | 3.93 | 54.67 | 52.82 | 13.64 | 9.82 | 1.00 |
| 4 | 4.00 | 90.00 | 50.00 | 13.47 | 9.95 | 1.00 |
| 5 | 3.39 | 84.17 | 51.54 | 13.50 | 9.78 | 1.00 |
| 6 | 3.49 | 76.76 | 49.27 | 13.64 | 9.83 | 1.00 |
| 7 | 3.35 | 75.82 | 58.63 | 13.95 | 9.95 | 1.00 |
| 8 | 3.95 | 54.58 | 66.67 | 13.71 | 9.91 | 1.00 |
| 9 | 3.77 | 65.04 | 52.98 | 14.08 | 10.10 | 1.00 |
| 10 | 3.33 | 73.94 | 58.89 | 13.95 | 9.92 | 1.00 |
| 11 | 3.76 | 78.43 | 52.99 | 14.13 | 10.21 | 1.00 |
| 12 | 3.47 | 67.64 | 56.21 | 14.03 | 10.00 | 1.00 |
| 13 | 3.51 | 82.44 | 54.59 | 13.88 | 10.02 | 1.00 |

**Table S9.** LC-MS/MS data of standard compounds

| **Phenolic compounds** | **R^2^** | **LOD** | **LOQ** | **Precursor m/z → Product m/z** | **Retention time (min)** |
| --- | --- | --- | --- | --- | --- |
| **Quercetin** | 0.99 | 2.03 | 6.76 | 301.00>151.20, 301.00>121.20, 301.00>107.10 | 7.81 |
| **Quercetin- 3-β-D-glucoside** | 0.99 | 2.32 | 7.74 | 462.80>301.10 | 6.66 |
| **Protocatechuic acid** | 0.99 | 1.48 | 4.93 | 153.00>109.10 | 5.60 |
| **Vitexin** | 0.99 | 2.04 | 6.79 | 430.80>311.20 | 6.36 |

**References**

1. Irakli M, Kleisiaris F, Kadoglidou K, Katsantonis D (2018) Optimizing extraction conditions of free and bound phenolic compounds from rice by-products and their antioxidant effects. Foods 7

2. Topuz S, Bayram M (2022) Oleuropein extraction from leaves of three olive varieties (Olea europaea L.): Antioxidant and antimicrobial properties of purified oleuropein and oleuropein extracts. J Food Process Preserv 46:1–12. https://doi.org/10.1111/jfpp.15697

3. Gaafar AA, Salama ZA (2013) Phenolic compounds from artichoke (Cynara scolymus L.) by-products and their antimicrobial activities. J Biol Agric Heal 3:e6

4. Re R, Pellegrini N, Proteggente A, et al (1999) Antioxidant activity applying an improved ABTS radical cation decolorization assay. Free Radic Biol Med 26:1231–1237. https://doi.org/10.1016/S0891-5849(98)00315-3

5. Blasi F, Urbani E, Simonetti MS, et al (2016) Seasonal variations in antioxidant compounds of Olea europaea leaves collected from different Italian cultivars. J Appl Bot Food Qual 89:

6. Benzie IFF, Strain JJ (1996) The ferric reducing ability of plasma (FRAP) as a measure of “antioxidant power”: The FRAP assay. Anal Biochem 239:70–76. https://doi.org/10.1006/abio.1996.0292

7. Topuz Türker S, Bayram M (2025) Phenolic Compound Profile, Antioxidant Capacity, and Physicochemical Characteristics of Commercial Hawthorn Vinegars. Akad Gıda 23:274–283. https://doi.org/10.24323/akademik-gida.1852506

8. Kim Y-M, Jeong Y-K, Wang M-H, et al (2005) Inhibitory effect of pine extract on α-glucosidase activity and postprandial hyperglycemia. Nutrition 21:756–761

9. Dambagı LY (2019) Antep fıstığı (Pistacia vera L.) meyve sapı ekstreleri ve saf metabolitlerinin antidiyabetik ve antikolinesteraz özellikleri. Kilis 7 Aralık Üniversitesi Fen Bilimleri Enstitüsü, Yüksek Lisans Tezi

.

**Acknowledgments** This research was financially supported by Tokat Gaziosmanpaşa University Scientific Research Projects Unit with the project No: 2022/38. The authors thanks to Tokat Gaziosmanpaşa University Scientific Research Projects Unit.

**Author contributions** EE: Methodology, experimental work, data curation, literature review, writing–original draft. STT: Methodology, experimental support, data analysis, literature review, writing–review & editing. MB: Conceptualization, study design, supervision. CK: Final proofreading and supervision.

**Funding** This work was supported by Tokat Gaziosmanpaşa University Scientific Research Projects Unit (TOGU-BAP) with the project No: 2022/38.

**Data availability** Data will be made available upon reasonable requests.

**Declarations**

**Conflict of interest** The authors declare no conflicts of interest.

**Ethics approval** This study does not need any ethics approval.
